# Effects of intraoperative inhaled iloprost on primary graft dysfunction after lung transplantation: A retrospective single center study

**DOI:** 10.1097/MD.0000000000003975

**Published:** 2016-07-08

**Authors:** Su Hyun Lee, Jin Gu Lee, Chang Yeong Lee, Namo Kim, Min-Yung Chang, Young-Chul You, Hyun Joo Kim, Hyo Chae Paik, Young Jun Oh

**Affiliations:** a Department of Anesthesiology and Pain Medicine, Anesthesia and Pain Research Institute; b Department of Thoracic and Cardiovascular Surgery; c Department of Radiology, Yonsei University College of Medicine, Seoul, Korea.

**Keywords:** iloprost, lung transplantation, primary graft dysfunction

## Abstract

**Design:**

Inhaled iloprost was known to alleviate ischemic-reperfusion lung injury. We investigated whether intraoperative inhaled iloprost can prevent the development of primary graft dysfunction after lung transplantation. Data for a consecutive series of patients who underwent lung transplantation with extracorporeal membrane oxygenation were retrieved. By propensity score matching, 2 comparable groups of 30 patients were obtained: patients who inhaled iloprost immediately after reperfusion of the grafted lung (ILO group); patients who did not receive iloprost (non-ILO group).

**Results:**

The severity of pulmonary infiltration on postoperative days (PODs) 1 to 3 was significantly lower in the ILO group compared to the non-ILO group. The PaO_2_/FiO_2_ ratio was significantly higher in the ILO group compared to the non-ILO group (318.2 ± 74.2 vs 275.9 ± 65.3 mm Hg, *P* = 0.022 on POD 1; 351.4 ± 58.2 vs 295.8 ± 53.7 mm Hg, *P* = 0.017 on POD 2; and 378.8 ± 51.9 vs 320.2 ± 66.2 mm Hg, *P* = 0.013 on POD 3, respectively). The prevalence of the primary graft dysfunction grade 3 was lower in the ILO group compared to the non-ILO group (*P* = 0.042 on POD 1; *P* = 0.026 on POD 2; *P* = 0.024 on POD 3, respectively). The duration of ventilator use and intensive care unit were significantly reduced in the ILO group (*P* = 0.041 and 0.038).

**Conclusions:**

Intraoperative inhaled iloprost could prevent primary graft dysfunction and preserve allograft function, thus reducing the length of ventilator care and intensive care unit stay, and improving the overall early post-transplant morbidity in patients undergoing lung transplantation.

## 1 Introduction

Primary graft dysfunction (PGD) is defined as an acute lung injury characterized by hypoxemia with pulmonary infiltration within 72 hours following lung transplantation and a main cause of early morbidity and mortality after lung transplantation, with 10% to 25% of recipients developing PGD after lung transplantation.^[1,2]^ The severity of PGD is classified by the International Society of Heart and Lung Transplantation on a scale of 1 to 3.^[3]^ In particular, PGD grade 3 is highly linked to post-transplant mortality^[4]^ and an increased risk of bronchiolitis obliterans.^[5]^ PGD grade 1 or 2 also prolongs mechanical ventilation and intensive care unit (ICU) stay, thereby potentially affecting the long-term outcomes.^[4]^ The pathogenesis of PGD involves multifactorial causes, including disruption of the integrity of the endothelial and alveolar epithelial barrier integrity,^[6]^ alteration of vascular permeability,^[7]^ and accumulation of neutrophil extracellular traps.^[8]^ Treatment of PGD involves conservative management of pulmonary edema, for example, using inhaled nitric oxide (NO),^[9,10]^ platelet-,^[11]^ complement-,^[12]^ and C-1 esterase inhibitors,^[13]^ although no definite therapeutic strategies for PGD have been established. Thus, with a rising interest in the prevention of PGD as a way to improve the post-transplantation outcomes, several clinical studies, including a meta-analysis, have been conducted to determine the risk factors of PGD after lung transplantation.^[14,15]^

Iloprost, a stable prostacyclin analog, helps maintain cyclic adenosine monophosphate (cAMP)-mediated intracellular calcium levels to promote pulmonary vasodilation in patients with pulmonary artery hypertension.^[16]^ Moreover, inhaled iloprost increases perfusion of well-ventilated pulmonary regions, reduces pulmonary shunt, and improves gas exchange,^[17]^ and iloprost has therefore been suggested as an alternative treatment to NO for acute respiratory distress syndrome.^[18]^ Furthermore, several animal studies have shown that iloprost-induced elevated intracellular cAMP acts to preserve the endothelial barrier in ischemia-reperfusion-induced acute lung injury,^[19]^ and inhaled iloprost as a pretreatment effectively improves surfactant function and attenuates postperfusion injury.^[20–22]^

To our knowledge, there are currently no clinical reports for the effects of inhaled iloprost on the prevention of PGD after lung transplantation. We aimed to investigate the short-term clinical efficacy of intraoperative inhaled iloprost on the prevention of PGD and improvement of allograft function.

## 2 Methods

### 2.1 Study design, setting, and participants

This study received approval from the Institutional Review Board of Severance Hospital (Ref. 4-2015-0952). Owing to the retrospective nature of the study, the need for informed consent was waived. Data for a consecutive series of patients who underwent lung transplantation with extracorporeal membrane oxygenation (ECMO), between January 2013 and January 2016, at Yonsei University, were retrieved.

A total of 85 patients who underwent lung transplantation with intraoperative ECMO were included in the analysis. By propensity score matching, 2 comparable groups of 30 patients were obtained: patients who received inhaled iloprost immediately after reperfusion of the grafted lung (ILO group); and patients who did not receive iloprost (non-ILO group). The Pulmonary Transplantation Council of the International Society of Heart and Lung Transplantation (ISHLT) has proposed revising the recipient selection criteria,^[23]^ which were last updated in 2006, and the recipient selection was based on these revised criteria. Patients in whom intraoperative ECMO was applied for cardiopulmonary support were included in this study. The cases of cardiopulmonary bypass or lung transplantation without cardiopulmonary support were excluded. Also, cases of single lung transplantation, >1 organ transplantation (including liver or kidney with lung transplantation), and combined operation of off-pump coronary artery bypass due to coronary artery disease were all excluded from this study. We obtained the follow-up information of donor and recipient by clinical chart review. The intraoperative and postoperative clinical data until postoperative days (PODs) 3 were recorded and analyzed.

### 2.2 Application of inhaled iloprost

Donor lungs were harvested after flushing with organ preservation solution (Perfadex: XVIVO, Göteborg, Sweden) and transported to the recipient's hospital. After anastomosis of the grafted bronchus, at the start of reperfusion of the lung, 2 mL (20 μg) iloprost (Ventavis: Bayer, Hamburg, Germany) was mixed with normal saline (3 mL) and aerosolized using an ultrasonic nebulizer (PARI BOY Sx: PARI GmbH, Starnberg, Germany) to each lung over 20 to 30 minutes. This nebulizer was connected to the inspiratory limb of the ventilator system. This nebulizer is connected to the inspiratory limb of the humidifier circuit, proximal to the patient. The circuit is approximately heated at 37°C to 38°C and maintained at this temperature throughout the lung transplantation. We also connected an air compressor to the nebulizer tubing. After attaching the nebulizer, we adjusted the tidal volume to 4 mL/kg to compensate for the bias flow. ECMO was maintained during inhalation of iloprost and thereby there was no abrupt onset of reduction of SpO_2_, tachycardia, or hypotension. After reperfusion of the grafted lung, protective ventilation management with low tidal volumes (positive end expiratory pressure, 5 cmH_2_O) was applied. The airway pressure was maintained at < 25 cm H_2_O, and the fraction of inspired oxygen (FiO_2_) was increased by 0.2 from 0.4 to maintain SpO_2_ ≥ 94%, as needed. In general, the patients were weaned off ECMO at the end of the operation. However, if there were signs of acute PGD or sustained pulmonary hypertension, ECMO was continued during the postoperative period.

### 2.3 Definition of PGD

The diagnosis of PGD was based on radiographic infiltrates consistent with pulmonary edema and reduction of the ratio of partial pressure of arterial oxygen to the fraction of inspired oxygen (PaO_2_/FiO_2_).^[3]^ According to this definition, recipients without radiographic pulmonary edema were graded as PGD 0, whereas those with radiographic pulmonary edema were graded as PGD 1, 2, or 3 if the PaO_2_/FiO_2_ ratio was > 300, 200 – 300, and < 200 mm Hg, respectively. Any patients who received ECMO after lung transplantation were designated as PGD grade 3.^[3]^

### 2.4 Definition of pulmonary infiltration

All chest radiographs, including those obtained in the first postoperative 72 hours, were reviewed in the chronological order by a radiologist who was blinded to the patient identification and demographic data. The severity of pulmonary infiltration was classified as minimal-diffuse infiltrate only, mild-diffuse infiltrate with consolidation (< 50% parenchymal involvement), moderate-diffuse infiltrate with consolidation (≥ 50% but < 90% parenchymal involvement), or severe-diffuse infiltrate with consolidation (≥ 90% parenchymal involvement) based on a previous study.^[24]^

### 2.5 Outcome

The primary outcome variable was defined as incidence of PGD within 72 hours after lung transplantation. The secondary outcome variable was duration of ventilator care. Also, intraoperative data, incidence of ECMO application after surgery, reapplication of ECMO within 3 days after weaning, duration of ICU stay and changes in the mean pulmonary artery pressure (mPAP) until PODs 3 were assessed.

### 2.6 Statistical analysis

Propensity score matching was performed to compensate for bias arising from the lack of randomization. One-to-one propensity score matching was conducted using a logistic regression with covariates of age, gender, and body mass index. Data were described by frequencies and percentages and were analyzed using the Kolmogorov–Smirnov test to assess for a normal distribution. Intergroup comparisons were analyzed using the Mann–Whitney *U* test or unpaired *t* test for continuous variables and the chi-square test or Fisher's exact test for categorical variables. Values are expressed as the means ± SD for continuous variables and as numbers (percentages) for categorical variables. Parameters showing a non-normal distribution are expressed as medians and interquartile ranges (IQR). Statistical analysis was performed using SPSS version 20.0 (SPSS Inc, Chicago, IL), with significance at *P* < 0.05.

## 3 Results

A total 84 patients underwent lung transplantation with ECMO were enrolled: 32 for ILO group and 52 for non-ILO group; and 2 patients with lung transplantation underwent concomitant cardiac surgery, such as off-pump coronary artery bypass, were excluded from the analysis. By propensity score matching, 30 matched groups were obtained. The donor and recipient characteristics were not significantly different between the 2 groups (Table 1**)**.

**Table 1:**
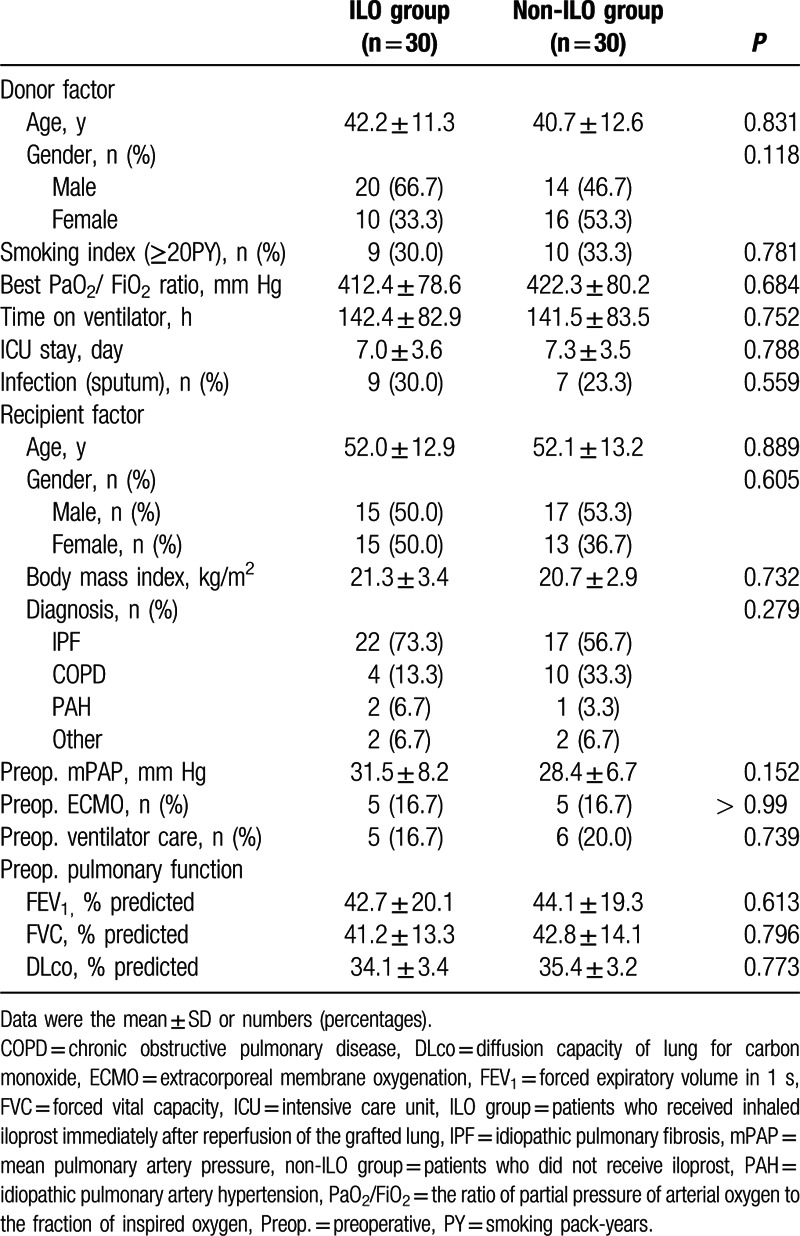
Donor and recipient factors.

As shown in Table 2, there was no significant difference in the intraoperative variables between the 2 groups. The overall incidence of applied ECMO until PODs 3 was significantly reduced in the ILO group compared to that in the non-ILO group. The durations of ventilator care and ICU stay were significantly shorter in the ILO group than in the non-ILO group. The mPAP on POD 3 was significantly reduced in the ILO group compared to that in the non-ILO group.

**Table 2:**
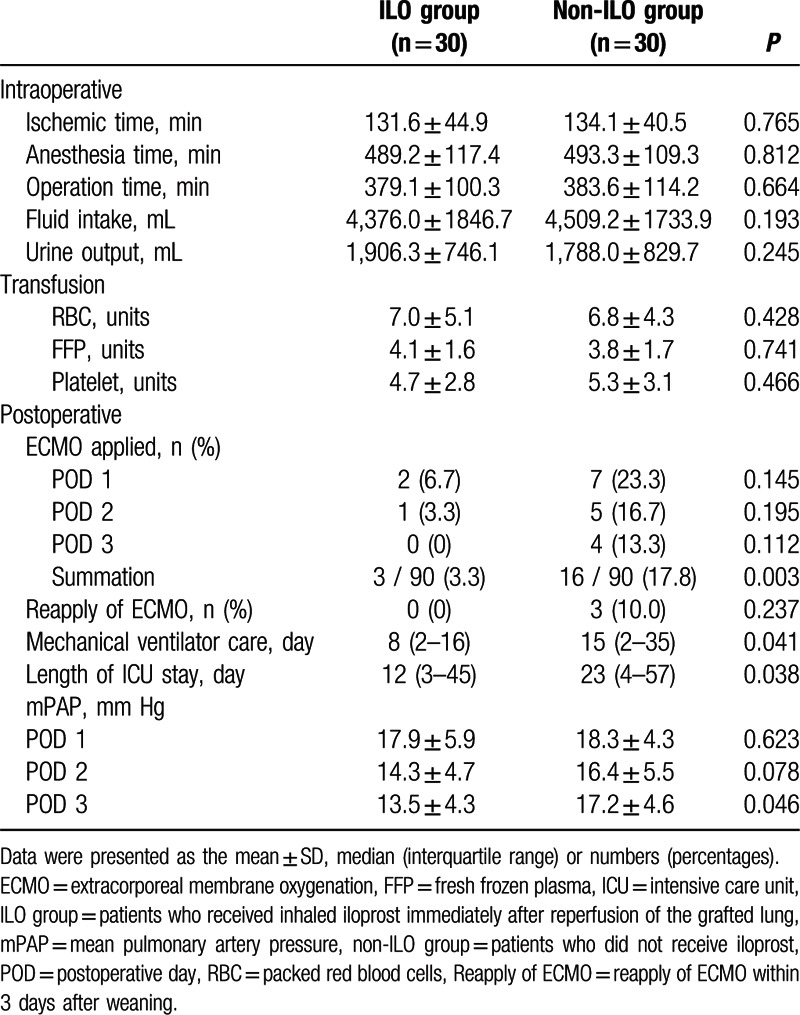
Perioperative data.

**Figure 1:**
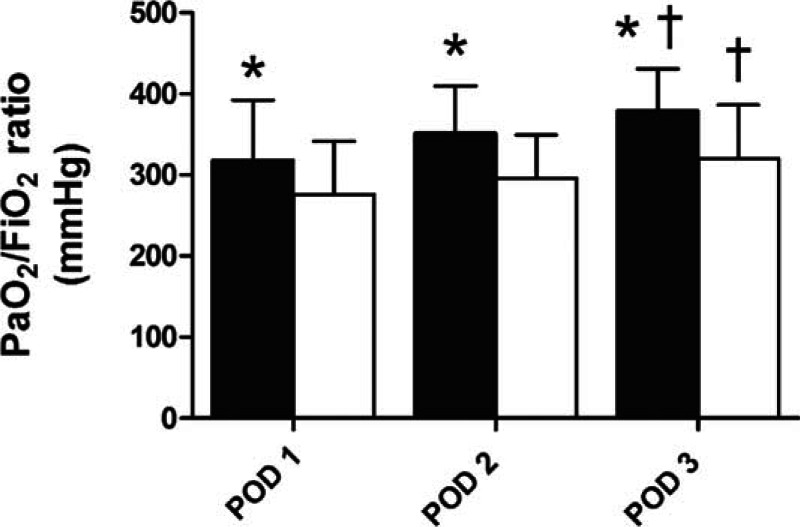
Changes in the PaO_2_/FiO_2_ ratio in 30 cases with intraoperative inhaled iloprost (black) and 30 subjects who did not receive inhaled iloprost (white) for 72 hours postoperatively. ^∗^*P* < 0.05 compared with non-ILO group; ^∗∗^*P* < 0.05 compared with POD 1. PaO_2_/FiO_2_ = the ratio of partial pressure of arterial oxygen to the fraction of inspired oxygen, POD = postoperative day.

The severity of pulmonary infiltration from POD 1 to 2 was significantly reduced in the ILO group compared to that in the non-ILO group (Table 3). In particular, minimal-to-mild pulmonary infiltration was more frequent in the ILO group than in the non-ILO group, whereas moderate-to-severe pulmonary infiltration was not present in the ILO group. As shown in Fig. 1, the PaO_2_/FiO_2_ ratio until PODs 3 was significantly higher in the ILO group than in the non-ILO group (318.2 ± 74.2 vs 275.9 ± 65.3 mm Hg, *P* = 0.022; 351.4 ± 58.2 vs 295.8 ± 53.7 mm Hg, *P* = 0.017; and 378.8 ± 51.9 vs 320.2 ± 66.2 mm Hg, *P* = 0.013, respectively). The overall incidence of PGD grade 3 was significantly lower in the ILO group, whereas the overall incidence of PGD grade 1 was significantly increased in the ILO group than in the non-ILO group until PODs 3 (Table 4).

**Table 3:**
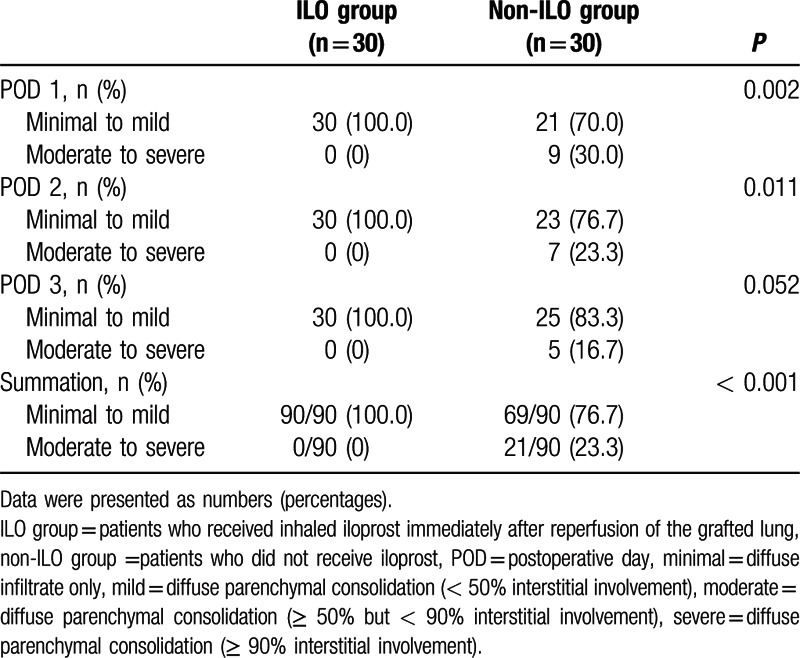
Severity of pulmonary infiltration for 72 h postoperatively.

**Table 4:**
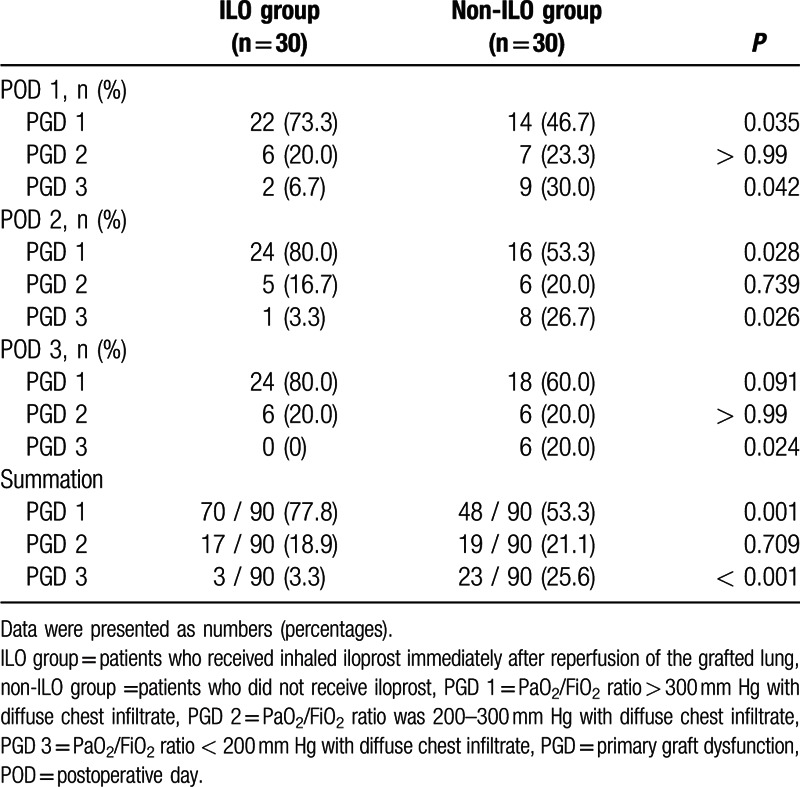
Grade of primary graft dysfunction.

## 4 Discussion

In this study, we report the novel finding that intraoperative inhaled iloprost, which is known to alleviate ischemic-reperfusion injury, improved the early postoperative PaO_2_/FiO_2_ ratios and lowered the severity level of pulmonary infiltration, thus decreasing the incidence of PGD grade 3 up to 72 hours post-lung transplantation. Consequently, inhaled iloprost administered immediately after reperfusion of the grafted lung may reduce the need for postoperative ECMO application and shorten the duration of ventilation care and ICU stay, as well as lower the overall early morbidity.

Although lung transplantation is an effective treatment for end-stage lung disease, the development of PGD increases postoperative morbidity and mortality. In particular, PGD grade 3 largely influences the 30-day survival rate^[1]^ and has been identified as a definite cause of early mortality, with a recent multicenter prospective cohort study reporting an incidence of 30.8% of PGD grade 3 in the first 3 days after lung transplantation.^[14]^ PGD is largely influenced by multiple risk factors, including various donor, recipient, and technical problems.^[14,25]^ Further, inflammation, which is augmented through ischemia-reperfusion injury, is now generally accepted to play a role in the development of PGD after lung transplantation.^[6,26]^ The inflammatory cascade induced by ischemia-reperfusion injury is a crucial factor in the development of PGD, suggesting that neutrophil extracellular traps are profusely present in recipient lungs with PGD.^[8]^ Several therapeutic agents, such as inhaled NO and platelet inhibitors, along with cytoprotective or anti-inflammatory agents, have been investigated to prevent PGD. Inhaled NO is a pulmonary vasodilator that increases cyclic guanosine monophosphate and attenuates immediate oxygenation during acute respiratory distress syndrome.^[27]^ However, some studies have reported that inhaled NO has no clinical efficacy in the prevention of PGD.^[9,10]^ Furthermore, as prolonged use of inhaled NO may have proinflammatory effects due to the nitrosylation of NO,^[28]^ it is difficult to expect any long-term effects. There is a recent interest in the association between plasma complement levels and PGD,^[29]^ with 1 clinical study indicating that C1-esterase inhibitor, a serine-protease-inhibitor that controls the complement pathway, may be effective in the treatment of PGD.^[13]^ However, that study differs from current study in the aspect that they indicated the clinical efficacy for the treatment of PGD rather than for the prevention. Another recent study reported that there was an association between the genetic variations of the prostaglandin pathway and development of PGD.^[30]^ Moreover, several experimental studies have provided evidence supporting the anti-inflammatory effects of iloprost against lung injury. A recent animal study demonstrated that iloprost preserved endothelial barrier regulation in an acute lung injury model and attenuated alveolar edema,^[19]^ and another study suggested that the prostanoid receptor signaling activated by iloprost has anti-inflammatory effects in mice.^[31]^ Additionally, previous animal studies have reported that inhaled iloprost attenuates the ischemia-reperfusion response of the donor lung and decreases postischemic pulmonary edema and alveolar leakage.^[21,22]^ Nevertheless, there are currently no clinical human studies that have demonstrated these effects and, to our knowledge, this is the first study to assess the clinical efficacy of intraoperative inhaled iloprost in patients undergoing lung transplantation.

Our results demonstrated that iloprost caused a shift in the severity of PGD (grade 3 to grade 1). Although previous studies have demonstrated that grade 1 is not associated with increased mortality,^[14,32]^ the reduced severity described in this study is expected, as iloprost is known to improve ventilation/perfusion matching.^[18]^ Inhaled iloprost diffuses through the lung parenchyma, inhibits infiltration, and decreases the incidence of pulmonary edema. In contrast, there is abundant literature in the field of critical care addressing the effects of inhaled epoprostenol on the improvement of oxygenation, but not mortality in patients with ARDS.^[33]^ The PGD and ARDS have similar mechanisms of lung injury resulting from a profound inflammatory process. However, the difference between previous studies and the current study is the time point of prostacyclin administration. In the current study, iloprost inhalation began immediately after reperfusion, which is the moment that the inflammatory process is activated. This process would not only improve hypoxemia but would also have anti-inflammatory effects and possibly prevent ischemia-reperfusion injury. Thus, iloprost inhalation as a preventative measure against the inflammatory process could reduce the incidence of PGD and decrease the length of the ICU stay.

Interestingly, moderate-to-severe pulmonary infiltration was not present within POD 3 in the ILO group in this study, as compared to in the non-ILO group. Starting from 1 hour after reperfusion, there are reportedly significant increases of superoxide, NO, and their reaction products, which participate in ischemia-reperfusion injury pathogenesis, and free radical bursts can be found in the peripheral blood 1 hour after the start of ischemia.^[34]^ Although the half-life of iloprost is only 20 to 30 min, the effects of cAMP induced by iloprost are maintained for 4 hours;^[35]^ therefore, we can postulate that inhaled iloprost administered directly after reperfusion of the grafted lung both suppressed the immediate response of lung ischemia-reperfusion injury and delayed the dissemination of radical bursts. During pulmonary vasodilation by means of inhaled iloprost, inhaled cAMP increases reabsorption of alveolar leakage, which decreases intra-alveolar edema and ameliorates dissemination of radical bursts, starting from perfusion of the grafted lung.^[36]^

There were several limitations to this study. This retrospective study was analyzed by a relatively small sample size of a single center, thus the informational bias cannot be entirely excluded. In this study, the same surgeons, who are proficient in cardiothoracic surgery, consistently performed the lung transplantations, and there was no significant difference in operation time or intraoperative blood loss. Subsequently, this may suggest that outcome assessment was not influenced by the learning curve.

Furthermore, the overall incidence of PGD grade 3 in the non-ILO group was slightly higher than the previously reported incidence. Pulmonary fibrosis, which is a known risk factor for PGD,^[15]^ is the most common etiology for the indication of lung transplantation in South Korea.^[37]^ Additionally, there is evidence suggesting that ECMO itself is a risk factor for PGD.^[38]^ It is possible that these factors aggravate the development of PGD. We only retrieved data from patients who underwent intraoperative ECMO. Recently, a study involving several large-volume centers implementing intraoperative ECMO during lung transplantation^[39]^ suggested ECMO as the treatment of choice in hemodynamically unstable patients. However, systemic inflammatory response syndrome, characterized by the development of a so-called cytokine storm, may develop in patients undergoing ECMO.^[40]^ Therefore, ECMO use might be a confounding factor in our study focusing on the effects of iloprost on PGD. The use of inhaled iloprost during lung transplantation must be based on evidence confirming the effects of iloprost in a randomized controlled trial. The authors of this study intend to conduct a prospective randomized trial to eliminate bias or confounding factor and evaluate the effects of inhaled iloprost on PGD. Also, we focused mainly on the occurrence of PGD, and the comprehensive analysis of early outcomes was limited.

In the current study, PGD was defined by radiographic infiltrates and PaO_2_/FiO_2_ as defined according to ISHLT criteria.^[3]^ Recently, plasminogen activator inhibitor type 1 (PAI-1) level and protein C levels are shown to be decreased in PGD grade 3.^[41]^ Because these biomarkers that reflect lung injury including plasma intercellular adhesion molecule 1, protein C, and PAI-1 are associated with PGD grade 3, it has been suggested that they may be included in defining PGD.^[42]^ Thus, not including assessment of these biomarkers is a limitation to this study and further trial may be needed to evaluate the effects of inhaled iloprost on the biomarkers.

In conclusion, despite the advancement in surgical techniques and proficient perioperative management, PGD remains a key factor in the early morbidity and mortality after lung transplantation. Control of inflammatory responses to ischemia-reperfusion injury is vital in the prevention of PGD. Nevertheless, our study suggested the efficacy of intraoperative inhaled iloprost as a preventative therapeutic intervention of PGD, and the long-term effects of inhaled iloprost have not yet been assessed. Accordingly, further randomized controlled studies focusing on the anti-inflammatory effects of intraoperative inhaled iloprost should be needed.
